# Nondestructive In-Situ Measurement of Alkaline Phosphatase Activity of Human Intestinal Organoids in Hydrogel Domes Using Scanning Electrochemical Microscopy

**DOI:** 10.1021/acs.analchem.5c07602

**Published:** 2026-03-06

**Authors:** Yoshinobu Utagawa, Ayaka Ogihara, Yasuhiko Shinoda, Fei Li, Hiroya Abe, Hitoshi Shiku, Kosuke Ino

**Affiliations:** † Graduate School of Engineering, 13101 Tohoku University, Sendai 980-8579, Japan; ‡ Organic Device Development Department, Material Development Division, Toyoda Gosei Co., Ltd., Ama 490-1207, Japan; § The Key Laboratory of Biomedical Information Engineering of Ministry of Education, School of Life Science and Technology, 12480 Xi’an Jiaotong University, Xi’an 710049, P. R. China; ∥ Bioinspired Engineering and Biomechanics Center (BEBC), Xi’an Jiaotong University, Xi’an 710049, P. R. China; ⊥ Frontier Research Institute for Interdisciplinary Sciences, Tohoku University, Sendai 980-8578, Japan

## Abstract

Human intestinal organoids serve as valuable models in regenerative medicine and drug screening. Nondestructive evaluation of both the hydrogel domes and the organoids cultured within them, including assessment of alkaline phosphatase (ALP), a key differentiation marker, remains a critical analytical challenge. To address this, we have developed a scanning electrochemical microscopy (SECM)-based approach that enables in-situ, real-time monitoring of ALP activity of organoids in hydrogel domes. Based on the recorded oxidation currents of enzymatic product of ALP, we have quantitatively determined ALP activity at the single-organoid level, reporting for the first time the enzyme activity in units per organoid (where 1 U corresponds to 1 μmol of product formed per minute). This innovative SECM strategy provides a noninvasive and spatially resolved analytical platform that is expected to advance organoid-based drug screening and transplantation studies.

## Graphical abstract

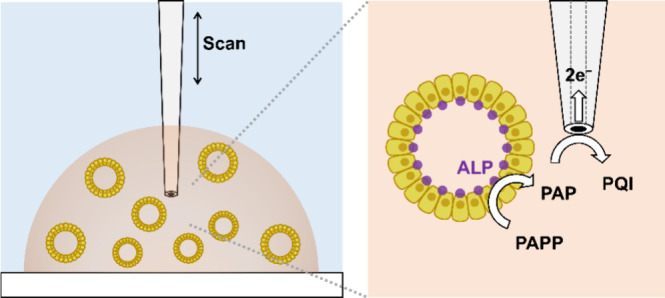

The intestinal epithelium plays a critical role in nutrient absorption and maintaining barrier function, with its dysfunction being associated with various diseases, such as inflammatory bowel disease.^1^ Accurately assessing the differentiation status and function of epithelial cells is therefore essential for both understanding their physiological functions and constructing disease models. While traditional two-dimensional cell cultures and animal models have been widely used, and in vitro models using Caco-2 cells have been widely reported,^2,3^ they fail to fully recapitulate the tissue structural complexity and cellular diversity of the native human intestinal epithelium.

Intestinal organoids, which are three-dimensional structures derived from stem cells, have emerged as a powerful model system for drug screening and basic research, because of their ability to mimic the self-organization of intestinal epithelium and formation of diverse cell lineages.^4^ Various techniques have been applied to engineering intestinal organoids.^5^ For instance, organoid-derived cells have been cultured on microfluidic platforms^6^ and biomimetic scaffolds.^7^ A critical requirement in building such systems is the ability to monitor organoid status-particularly during key phases such as proliferation-without disrupting their structural integrity, in order to ensure experimental reproducibility and reliable quality control. To meet this need, organoids are commonly cultured within hydrogel domes (e.g., Matrigel) to support their three-dimensional architecture.

Current methods for assessing live organoid function face some limitations. For example, biochemical assays for gene or protein expression typically require dissolution of the hydrogel domes. While live-cell imaging using fluorescent probes allows for nondestructive evaluation of processes,^8^ such as transport^9^ and barrier functions,^10^ these optical methods generally depend on external labeling, which may perturb native physiology of cells. As a label-free alternative, electrochemical techniques have been demonstrated to evaluate live organoid function via real-time monitoring of signaling molecules, metabolites and enzyme activities. For instance, electrochemical monitoring of serotonin released from intestinal organoids in hydrogel domes has been realized using electrochemical multiwell plates with placing electrodes outside hydrogel domes.^11^ Despite these examples, a major technique challenge lacks the capability to perform localized, in-situ quantitative measurements within the dome at the level of individual organoids remains, which is crucial for assessing organoid heterogeneity and enabling precise, single-organoid analysis in future pharmacological studies.

To address this gap, we developed a scanning electrochemical microscopy (SECM)-based approach that enables spatially resolved, in-situ quantitative measurement of enzyme activity within intestinal organoids, without damaging the hydrogel dome or requiring genetic labels (Figure 1). This method allows direct noninvasive mapping and quantification of alkaline phosphatase (ALP) activity—a key differentiation marker^12^—at the single-organoid level. The nondestructive feature allows repeated monitoring of the ALP activity within the same dome, which is a novel capability. This SECM platform provides a new analytical tool that advances organoid-based research and drug development.

**Figure 1:**
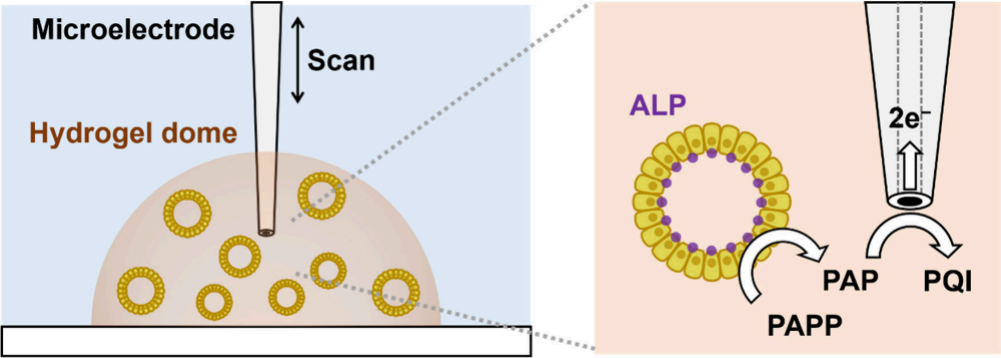
Schematic of electrochemical measurements of ALP in intestinal organoids cultured in hydrogel domes. PAPP was converted to PAP by ALP, and PAP was oxidized to PQI on an SECM probe. The microelectrode was positioned adjacent to the organoids and scanned vertically in 300 μm up-and-down movements, repeated for three cycles.

SECM is a type of scanning probe microscopy equipped with a microelectrode and is widely used to evaluate respiratory^13–16^ and enzyme activities^17^ of cells. It has also been used to analyze spheroids^18,19^ and cells embedded in 3D-printed hydrogel.^20^ In addition, we previously reported SECM measurements of respiratory activity in cancer organoids in solution.^21^ In this study, an SECM probe was inserted into hydrogel domes containing intestinal organoids and positioned the microelectrode near an organoid. This approach enables nondestructive measurements, because of the small size of the probe, which offers a clear advantage over conventional approaches for organoid analysis. As a proof of concept, ALP activity was evaluated electrochemically using *p*-aminophenyl phosphate (PAPP) as the substrate (Figure 1). Previous studies have established that PAPP is converted to *p*-aminophenol (PAP) by ALP, and PAP is oxidized to *p*-quinone imine (PQI) on an electrode.^22,23^ These systems evaluate the ALP activity of entire two-dimensional or three-dimensional culture areas using a single working electrode placed on a chip device by monitoring the activity of all cells on the sensor; however, electrochemical evaluation of ALP activity at individual single cells or cell aggregates has not yet been achieved because the redox currents from the integrated electrode cannot be separated for individual analysis. In contrast, previous SECM works have evaluated ALP activity of individual spheroids,^24^ but SECM studies focusing on the activity of organoids in hydrogels have not yet been systematically discussed, because probe insertion into hydrogels and electrochemical reactions within hydrogels may affect SECM signals and the hydrogel itself. Nevertheless, hydrogel culture is essential for organoid formation. This study presents an SECM approach as a nondestructive in-situ method for evaluating ALP activity in single intestinal organoid inside hydrogel domes, enabling quantitative analysis of ALP activity (U per organoid) based on scanning data and spherical diffusion theory, which provides a clear advantage over fluorescence and absorbance methods.

The preparation of the hydrogel domes is described in the Supporting Information. Briefly, 60 μL of Matrigel was placed in a 24-well plate on a plate heater at 37 °C. After 5 min, the plate was placed upside down and incubated for 15 min, resulting in hydrogel domes. The effects of electrochemical measurement conditions on the stability of the Matrigel-based hydrogel domes were investigated with respect to temperature, pH, and the presence of enzyme substrates (Figure 2A). The stability at 25 and 37 °C was evaluated because the procedures include steps at both room temperature (25 °C) and in an incubator. Additionally, the effect of PAPP on stability was investigated. These conditions did not affect dome stability. However, the domes disappeared after 1 h under alkaline conditions (pH 9.0). This is because alkaline conditions disrupt ionic and hydrogen bonds in proteins, causing the structure to break down. In contrast, the domes were stable at physiological pH (pH 7.4). Therefore, we selected pH 7.4 and 37 °C as the conditions for measuring ALP activity in the domes. Because the expression of acid phosphatase^25,26^ is low in the intestinal epithelium and ALP is active at physiological pH,^27^ it was assumed that the electrochemical signals primarily originate from ALP. Subsequently, a calibration curve for PAP in the domes was prepared. The electrochemical methods are described in the Supporting Information. Briefly, the hydrogel domes were immersed in Dulbecco’s phosphate-buffered saline (PBS) containing 4.7 mM PAP. A Pt microelectrode (Φ = 20 μm) was inserted into the domes, and a Ag/AgCl (sat. KCl) reference electrode was placed in the solution. The electrodes were then connected to a potentiostat (HV-405 SECM system). In this study, 0.3 V was chosen because PAP can be selectively monitored even in the presence of PAPP (Figure S1). After immersing the hydrogel domes in the solutions containing each PAP concentration for 30 min, the oxidation currents of PAP were measured by applying 0.3 V to the microelectrode. Figure 2B shows the average oxidation current of PAP during three scans with 300 μm upward and downward movement in the hydrogel domes. The oxidation currents corresponded to the PAP concentrations. However, the sensitivity was lower than that measured in solution (Figure S2). This result suggests that electrode fouling may have reduced the sensitivity, as the electrode was in contact with the Matrigel for approximately 30 min. Although electrode fouling reduced sensitivity, electrochemical measurements of PAP could be performed in the hydrogel domes. The calibration curve was used for the flux analysis of ALP activity. The diffusion coefficient of PAP was determined to be 7.55 × 10^–10^ m^2^/s, based on the slope shown in Figure S1B, using the theory described in the Supporting Information.

**Figure 2:**
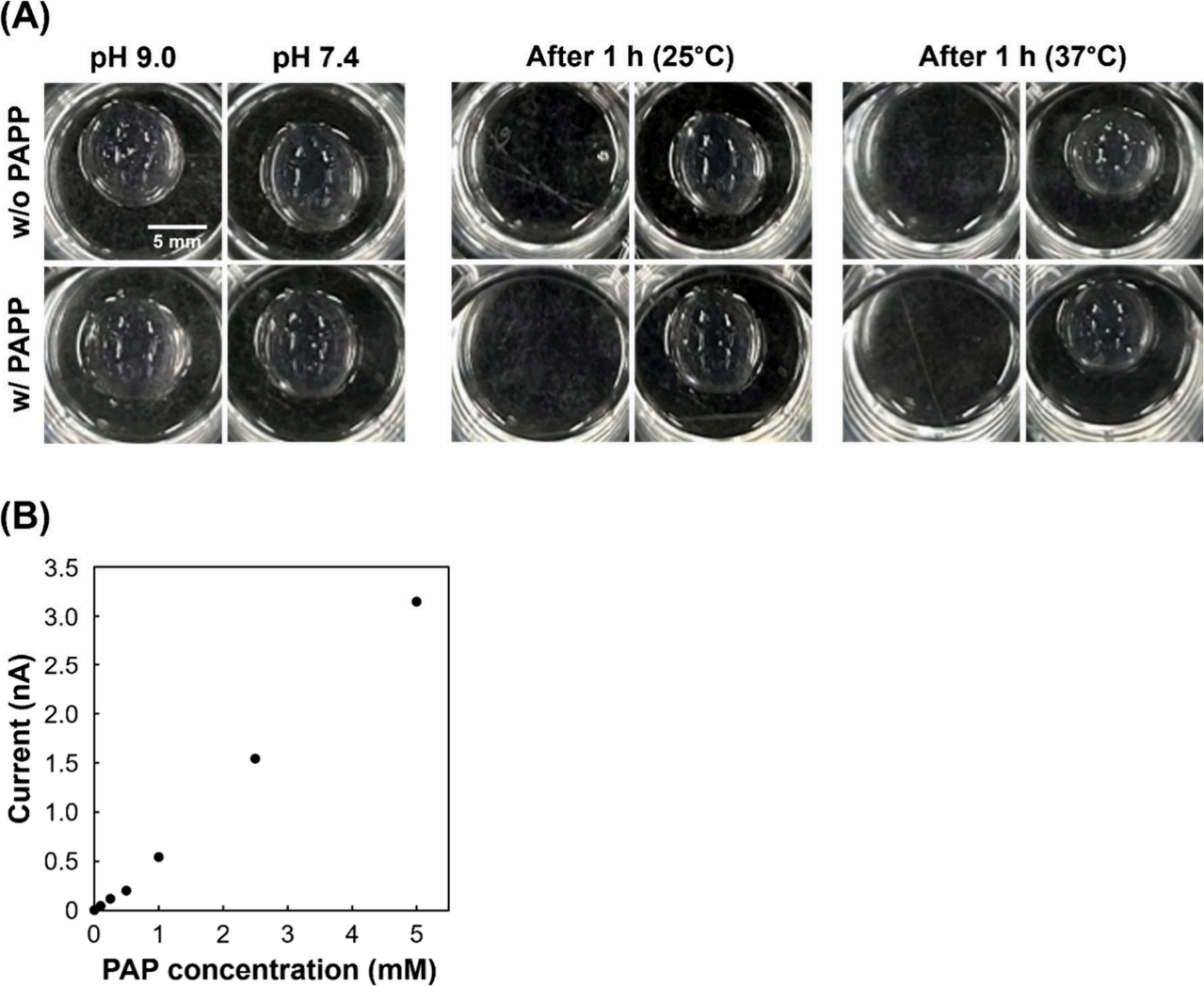
Characterization of hydrogel domes under electrochemical measurement conditions. (A) Effects of pH (7.4 and 9.0) and 4.7 mM PAPP on hydrogel dome constructs. Alkaline conditions disrupted the domes within 1 h, whereas neutral pH, PAPP, and temperature had no effect. (B) Calibration curve of PAP in domes. Potential = 0.3 V.

The ALP activity in multiple organoids in the hydrogel domes was assessed. The cell culture methods are described in the Supporting Information. Human intestinal organoids (Def-INTESTINAL; DefiniGEN, UK) were cultured in Matrigel domes. The organoids self-organized to form lumens (Figure S3). The ALP activity in organoids cultured for 5 days was measured electrochemically. Briefly, the culture medium was replaced with PBS (pH 7.4) containing 4.7 mM PAPP at 37 °C. The microelectrode was inserted into the domes containing the organoids and the reference electrode was placed in the solution. After 30 min, 0.3 V was applied to the microelectrode, and the electrode was scanned three times. Figure 3A shows an image of the multiple organoids and the microelectrode in the dome. In this measurement, 16–125 organoids were closely packed in each dome. Intestinal organoids form hollow structures. If a smooth monolayer is formed and the cell diameter is ∼10 μm, the cell number is estimated to be on the order of ∼(1–2) × 10^3^ cells in a 200-μm-diameter intestinal organoid. As shown in Figure 3B, oxidation currents were obtained from organoids in the presence of the enzyme substrate, indicating that ALP activity was successfully measured. The z position of the SECM probe did not significantly affect the oxidation current (Figure S4), indicating that the enzymatic products were uniformly distributed in the domes, as the diffusion layers of the enzymatic products around each organoid overlapped. This result indicates that crowded domes can be treated as an effective compartment. It was necessary to adjust the organoid concentration to enable single-organoid analysis. In contrast, the oxidation currents increased as the number of organoids in the domes increased, indicating that this approach is quantitative for ALP analysis in organoids. Moreover, the hydrogel dome structure did not change before or after the measurements, indicating that SECM evaluated the organoids cultured in hydrogel domes without disrupting them (Figure 3C). Therefore, this system can be used to measure the ALP activity of the same organoids multiple times while maintaining a continuous culture.

**Figure 3:**
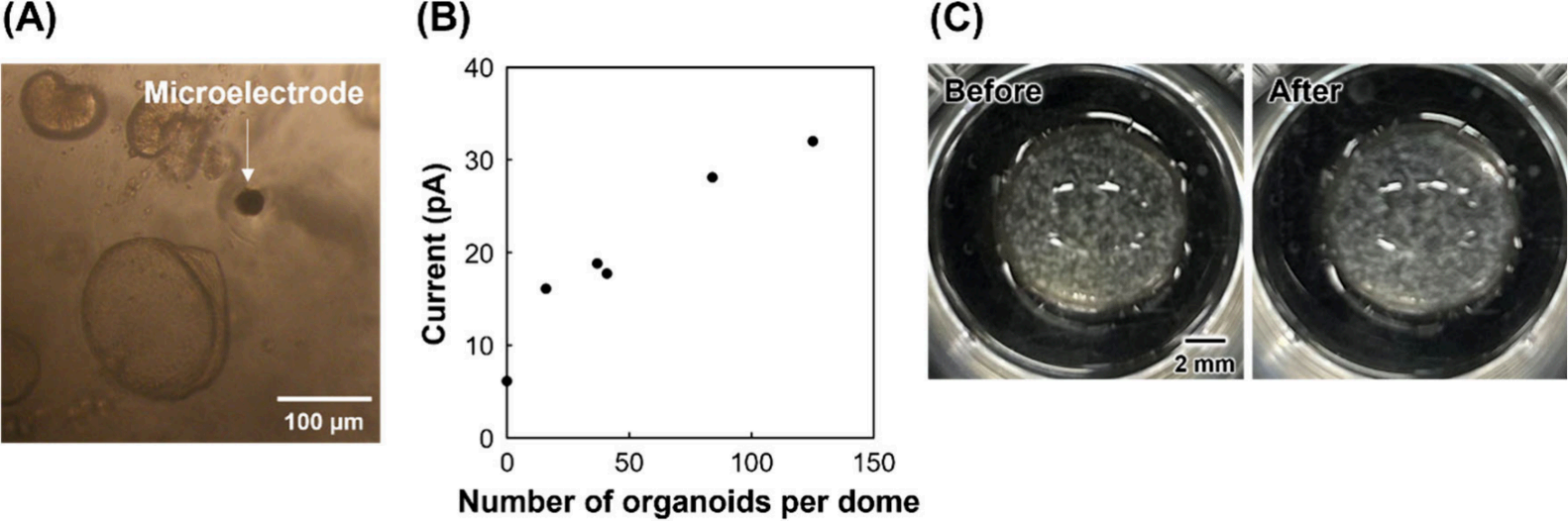
SECM of ALP activity in multiple intestinal organoids within hydrogel domes. Potential = 0.3 V. Culture period = 5 days. (A) Multiple organoids within hydrogel domes. (B) Current vs number of organoids per dome. (C) Hydrogel domes before and after SECM probe insertion.

Next, we performed multiple measurements on the same domes. Initially, damage to the organoids caused by the measurements was investigated. Organoids were treated with a solution containing 4.7 mM PAPP for 1 h and analyzed using SECM. Live/dead staining revealed that most cells were alive after measurements (Figure 4A and 4B). The organoids continued to grow by day 5, even though they had been measured on day 3 by SECM (Figure 4C), suggesting that the proposed approach did not significantly affect organoid culture within the hydrogel domes. Furthermore, the amount of ALP per organoid increased from day 3 to day 5 as the organoids increased in size (Figure 4D). Because a growth medium was used in this study, the ALP activity exhibited only a moderate increase. However, when a differentiation medium is used to induce differentiation into enterocytes,^12^ ALP activity is expected to increase dramatically.

**Figure 4:**
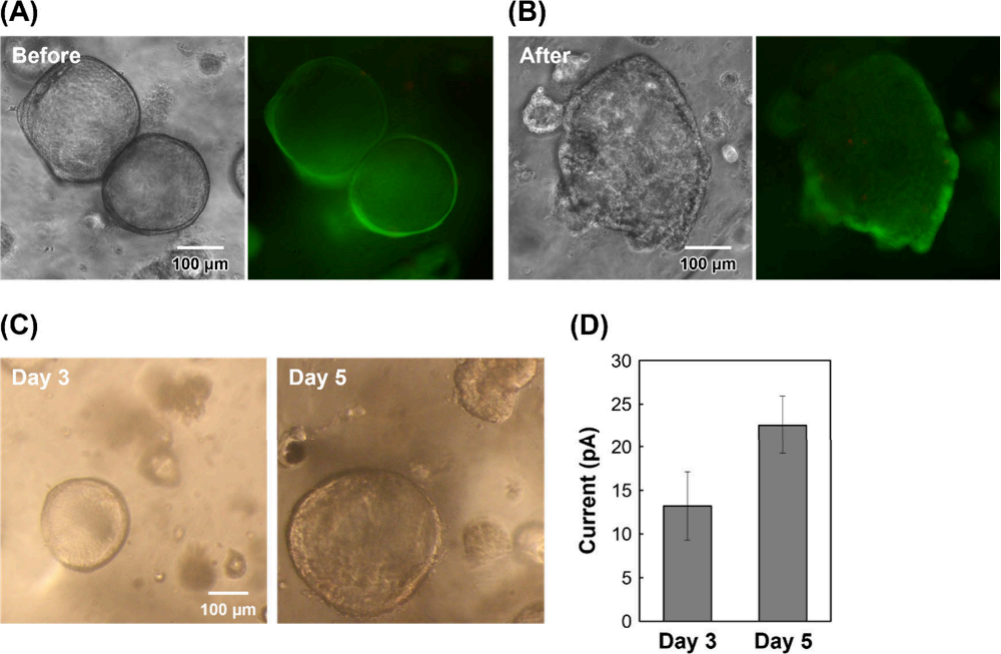
Cell viability and ALP activity in multiple intestinal organoids using the same domes on days 3 and 5. Phase-contrast and fluorescence images of organoids on day 5 (A) before SECM and (B) after SECM. Green: living cells; red: dead cells. (C) Images of organoids cultured for 3 and 5 days. (D) Current vs culture period (*n* = 3). Error bars represent standard deviations.

Next, the ALP activity of a single isolated organoid within the hydrogel domes was measured. Evaluating ALP activity at the single-organoid level enables the selection of organoids with comparable differentiation states, which improves data reproducibility. When the domes contained many organoids, the diffusion of PAP produced by each organoid was interfered. In contrast, the analysis can be based on local spherical diffusion of PAP around isolated organoids. The microelectrode was positioned adjacent to the organoid (Figure 5A). The organoid was at least 600 μm away from other organoids (Figure S5). Figure 5B shows the current responses during three scans of a single organoid. Oxidation current gradients were observed during the approaches and retractions. The subtracted current was calculated to remove the background current, and then the PAP concentration gradient was calculated using the calibration curve in Figure 2B (Figure 5C). Parameters *r*_s_ and *L* indicate organoid radius and distance between the center of the organoid and the electrode, respectively (Figure S6). The PAP production rate of the single spheroid was calculated using the spherical diffusion theory described in the Supporting Information. Briefly, *I* vs *r*_s_/*L* was plotted, and the slope was used for calculation of the production rate. The PAP production rate of a representative single organoid was 9.22 fmol/s per organoid. Here, the diffusion coefficient in the domes was assumed to be the same as that in solution because the diffusion coefficient in the domes could not be determined due to the electrode contamination. Therefore, the actual PAP production rate is expected to be lower, because the diffusion coefficient in the domes is smaller than that in solutions. As 1 U equals 1 μmol of product formed per min, the ALP activity per organoid was calculated to be 0.553 μU/organoid. According to previous studies, the ALP activity of Caco-2 cells cultured in a monolayer under static conditions for 21 days is approximately 0.1–10 μU/cell.^28,29^ The expression of ALP in organoids may be low due to insufficient differentiation. The difference in enzyme activity could also be partly due to differences in reaction kinetics arising from variations in pH during treatment.

**Figure 5:**
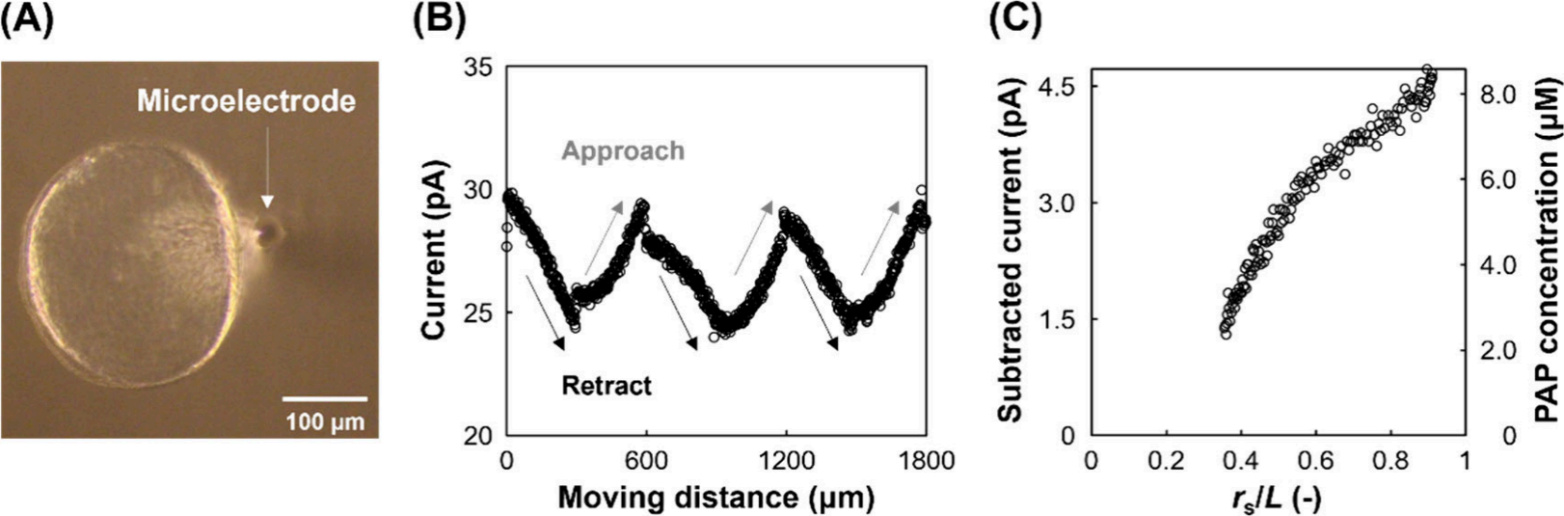
SECM of ALP activity in a single isolated intestinal organoid. Potential = 0.3 V. Culture period = 5 days. (A) Single isolated organoid. (B) Current responses during three 300 μm up-and-down scans near the organoid. (C) Plots of subtracted current (left axis) and PAP concentration (right axis) vs *r*_s_/*L*.

Although the diffusion coefficient of PAP in Matrigel has not been reported, diffusion coefficients of small molecules such as rhodamine B in water and Matrigel have been reported as 4.2 × 10^–10^ and 3.32 × 10^–10^ m^2^/s, respectively.^30,31^ According to these studies, the diffusion coefficient of PAP, a small molecule, in Matrigel is estimated to be approximately 80% of that in bulk solution. If this assumption is correct, the ALP activity would decrease to approximately 80% of the originally calculated value, since it is linearly proportional to the diffusion coefficient (eq S3). For a more accurate evaluation, the diffusion coefficient in the Matrigel dome should be experimentally determined in future work.

Finally, the diffusion of PAP from the organoids was simulated to confirm the overlap of the diffusion layers rather than to reproduce absolute currents. When organoids were separated by 200 μm, the diffusion layers overlapped (Figure S7A). Organoids were isolated by separating them by over 800 μm (Figure S7B). Therefore, individual organoids can be measured when they are separated by more than 800 μm. Furthermore, even if the organoids are densely packed, signals from individual organoids may be separated by performing scans at multiple locations, and the data can potentially be analyzed using AI and computational methods, provided that concentration profile data are available. SECM is an excellent method because it can be used to measure concentration profiles.

In conclusion, we reported an SECM-based methodology for quantitative, in-situ assessment of ALP activity in human intestinal organoids cultured within hydrogel domes. We first identified the stability of Matrigel domes, which necessitated measurements at physiological pH (7.4). Subsequently, we successfully quantified the ALP activity of organoids within entire domes and, more significantly, achieved the first spatially resolved measurement at the single-organoid level. The PAP production rate of a single organoid was 9.22 fmol/s, assuming the same diffusion coefficient in the hydrogel as in solution. This represents the first report of SECM being used to determine enzyme activity in units per organoid from human intestinal organoids. By providing a direct, label-free link between individual organoid morphology and its biochemical function, this SECM platform opens new avenues for selecting high-quality organoids based on functional metrics, a capability beyond the scope of conventional microscopy. This capability promises to enhance the precision and reliability of organoid-based drug screening and transplantation therapies.

## Supplementary Material



## Data Availability

All data presented in this study are available upon reasonable request to the corresponding author.

## Notes

Materials and methods, cyclic voltammograms of 1 mM PAP and 1 mM PAPP, calibration plot of PAP, images of organoids, SECM of ALP activity in various organoids, image of Figure 4A before trimming, schematic for calculating PAP concentration, simulation of diffusion (PDF)
