# Supplementary material for: Nondestructive In-Situ Measurement of Alkaline Phosphatase Activity of Human Intestinal Organoids in Hydrogel Domes Using Scanning Electrochemical Microscopy
Source: Anal Chem. 2026 Mar 6;98(12):8804–9. doi: 10.1021/acs.analchem.5c07602 (PMC13044878; doi:10.1021/acs.analchem.5c07602)
Supplement: Supplementary file 1 [file ac5c07602_si_001.pdf]

Supporting Information

Non-destructive in-situ measurement of alkaline phosphatase activity of human intestinal organoids in hydrogel domes using scanning electrochemical microscopy

Yoshinobu Utagawa,[a] Ayaka Ogihara,[a] Yasuhiko Shinoda,[b] Fei Li,[c,d] Hiroya Abe,[a,e] Hitoshi Shiku*[a] and Kosuke Ino**[a]

[a] Graduate School of Engineering, Tohoku University, Sendai 980-8579, Japan

[b] Organic Device Development Department, Material Development Division, Toyoda Gosei Co., Ltd., Ama 490-1207, Japan

[c] The Key Laboratory of Biomedical Information Engineering of Ministry of Education, School of Life Science and Technology, Xi'an Jiaotong University, Xi'an 710049, P. R. China.

[d] Bioinspired Engineering and Biomechanics Center (BEBC), Xi'an Jiaotong University, Xi'an 710049, P. R. China.

[e] Frontier Research Institute for Interdisciplinary Sciences, Tohoku University, Sendai 980-8578, Japan

*,** Corresponding authors:

E-mail address: hitoshi.shiku.c3@tohoku.ac.jp, and kosuke.ino@tohoku.ac.jp

Contents

-Materials and methods

-Fig. S1: Cyclic voltammograms of 1 mM PAP and 1 mM PAPP

-Fig. S2: Calibration plot of PAP

-Fig. S3: Images of organoids

-Fig. S4: SECM of ALP activity in various organoids

-Fig. S5: Image of Fig. 4A before trimming

-Fig. S6: Schematic for calculating PAP concentration

-Fig. S7: Simulation of diffusion

Materials and Methods

Cell culture

Human intestinal organoids (Def-INTESTINAL; DefiniGEN, UK) were embedded in Matrigel (Corning, USA) cultured in advanced Dulbecco's modified eagle medium (DMEM)/F12 (Thermo Fisher Scientific, USA) containing penicillin/streptomycin (PS; Gibco, USA), L-glutamine (Thermo Fisher Scientific), B-27 (Thermo Fisher Scientific), N2 supplement (Thermo Fisher Scientific), and HEPES (Sigma Aldrich, USA), which is Def-INTESTINAL thawing, recovery, and maintenance medium (IRMM). In addition to IRMM, Def-INTESTINAL Supplement Additive A (IRMM-A) was also used, containing R-spondin (Qkine Ltd., UK), noggin (R&D Systems, Inc., USA), prostaglandin E2 (Cayman Chemical Company, USA), EGF (R&D Systems, Inc.), Chiron (Selleck Chemicals LLC, USA), and A83-01 (Tocris Bioscience, UK). Cells were maintained in a humidified atmosphere containing 5% CO_2 at 37°C.

Initially, 200 μL of IRMM, 210 μL of Matrigel, and 0.7 μL of IRMM-A were mixed on ice. Subsequently, 60 μL of the solution was added to each well of a 24-well plate. The plate was incubated for 5 min on a plate heater. After gelation, the plate was incubated upside down for 15 min, 1 h after dome formation.

Domes containing organoids were washed with PBS. Subsequently, 500 μL of cold recovery solution was added to the wells, and the domes were incubated at 4°C for 30 min. After the Matrigel had dissolved, the solution was collected in a centrifuge tube and allowed to stand for 5 min. The supernatant was discarded, and the organoids were washed with 10 mL of medium. The organoids were centrifuged at 200 × *g* for 2 min, and the supernatant was discarded. Following this, 1 mL of medium was added, and the organoids were mechanically dissociated by pipetting. Subsequently, 5 mL of additional medium was added, and the organoids were centrifuged at 200 × *g* for 2 min. The supernatant was discarded, and the pellet was resuspended in 390 μL of Matrigel, 1.3 μL of IRMM-A, and 0.39 μL of ROCK inhibitor. At this stage, the organoid concentration was reduced to approximately one-third to one-fourth. Following this, 60 μL of the solution was dispensed onto a pre-warmed 24-well plate to form domes. After gelation, 700 μL of IRMM-A medium and ROCK inhibitor was added to each well. The medium was changed on days 3 and 5 after thawing, and the organoids were passaged on days 6 or 7.

Evaluation of hydrogel dome stability

Initially, 700 μL of 4.7 mM *p*-aminophenyl phosphate (PAPP; LKT Laboratories, Inc., USA) in Tris–HCl (pH 9.0) or PBS (pH 7.4) was added to each well. The domes were incubated at room temperature (25°C) and at 37°C in an incubator for 1 h. After incubation, the domes were observed.

Electrochemical measurements of PAP in the hydrogel domes

Initially, 700 μL of 0–5 mM *p*-aminophenol (PAP; Wako Pure Chemical Industries, Ltd., Japan) in Tris–HCl (pH 9.0) or PBS (pH 7.4) was added to the wells containing the domes at 37°C. A Pt microelectrode (Φ 20 μm) was inserted into the dome, and a Ag/AgCl (sat. KCl) electrode was placed in the solution. The electrodes were connected to a potentiostat (HV-405 SECM system). After 30 min, 0.3 V was applied to the microelectrode, and electrochemical measurements were performed. The same measurements were performed in the solution without domes. The diffusion coefficient (D (m^2/s)) was calculated using the following equation[1]:

$$I = 4nFCDa \qquad \text{(S1)}$$

where I is the limiting current, n is the number of electrons transferred ($n = 2$), F is the Faraday constant (96,485 C/mol), C is the PAP concentration, and a is the disk radius of the electrode (a = 10 μm).

SECM measurements of ALP activity of a single organoid

Intestinal organoids were cultured for 3–5 days in the domes. Following this, 700 μL of PBS (pH 7.4) containing 4.7 mM PAPP was added to the wells with domes containing organoids at 37°C. A microelectrode was placed in the dome, and a reference electrode was placed in the solution. The electrodes were connected to a potentiostat. After 30 min, 0.3 V was applied to the electrode, and it was scanned vertically 300 μm upward and downward at a speed of 20 μm/s; this scan was repeated three times.

The electrode surface was polished before each SECM measurement. When the redox signals were clearly abnormal or the probe was damaged, a fresh electrode was used after confirming its performance with ferrocenemethanol.

From Eq. S1, the limiting current is proportional to the concentration. Therefore, the concentration gradient can be calculated using the current gradient. In this study, the subtracted current was calculated assuming that the PAP concentration in the bulk solution was zero. The subtracted current value was obtained by subtracting the calculated bulk current from the current at each point. PAP concentration was then calculated using the calibration curve and the subtracted currents.

The PAP concentration near an organoid (C (mol/m^3 = mM)) was calculated based on Eq. S2 according to spherical diffusion theory[2]:

$$C = C_s \frac{r_s}{L} \qquad \text{(S2)}$$

where C_s (mol/m^3 = mM) is the PAP concentration at the surface of an organoid, r_s (m) is the organoid radius, and L (m) is the distance between the center of the organoid and the electrode. Eq. S2 can be used under steady-state conditions. As mentioned in the main text, the diffusion coefficient of PAP in

Matrigel is assumed to be 6.0 × 10^{-10} m^2/s. The time required for PAP to reach a distance of approximately 2–5 times the diameter of an organoid (200 µm) from its center was estimated to be 1–7 min. Thus, the steady-state analysis can be performed in this study, since the incubation time is 30 min.

The rate of PAP production per organoid (F (mol/s)) was calculated as follows:

$$F = 4\pi r_s D C_s \qquad \text{(S3)}$$

where D (m^2/s) is the diffusion coefficient of PAP (7.55 × 10^{-10} m^2/s in bulk solution).

The diameter of the probe tip (approximately 30 µm) is much smaller than that of an organoid (e.g., 200 µm), indicating that the probe is unlikely to disturb the formation of the diffusion layer. Therefore, a purely spherical diffusion model can be applied for the analysis. In addition, since the scan data were used to calculate ALP activity (Fig. 5C), the effect of probe proximity is negligible.

In this study, the effects of the boundary between the hydrogel dome and the surrounding aqueous solution were not considered to avoid complex analyses. In this study, the organoid was 600 µm away from the boundary.

Live/dead staining

Organoids cultured in the domes were measured using SECM. The organoids were fully exposed to the PAPP solution for 1 h. After the measurements, the solution was replaced with culture medium, and the organoids incubated for 3 h. The organoids were stained using a double staining kit (Dojindo, Japan) for 30 min at 37°C. Live (green) and dead (red) cells were observed under a fluorescence microscope (ECLIPSE Ti2; Nikon, Japan).

References

1. Bard, A. J.; Denuault, G.; Lee, C.; Mandler, D.; Wipf, D. O., Scanning Electrochemical Microscopy - A New Technique for the Characterization and Modification of Surfaces. *Acc. Chem. Res.* **1990,** *23*, 357-363.

2. Ino, K.; Mashiko, M.; Kanno, Y.; Tang, Y.; Masui, S.; Nisisako, T.; Hiramoto, K.; Abe, H.; Shiku, H., Extended Spherical Diffusion Theory: Electrochemiluminescence Imaging Analysis of Diffusive Molecules from Spherical Biosamples. *Anal. Chem.* **2024,** *96*, 18967-18976.

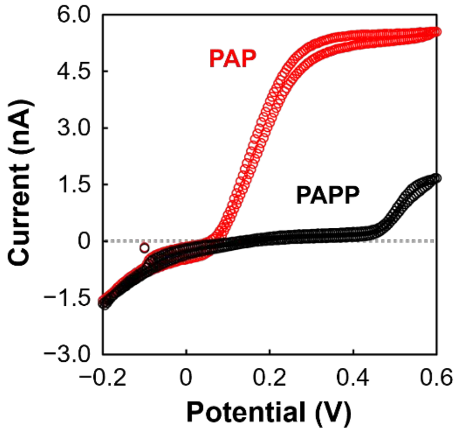

Fig. S1

Cyclic voltammograms of 1 mM PAP and 1 mM PAPP in pH 7.4 solutions. Scan rate: 50 mV/s.

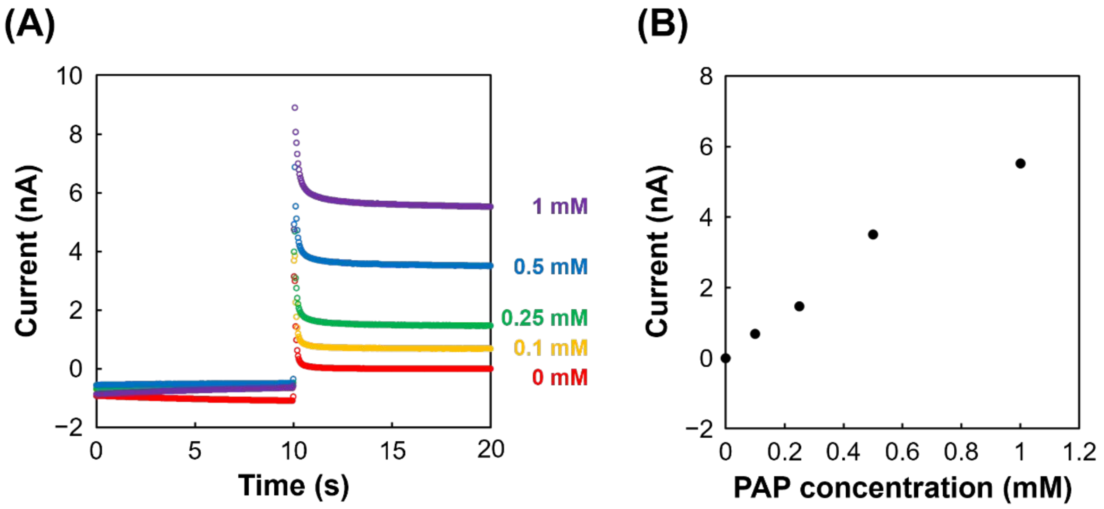

Fig. S2

Electrochemical measurements of PAP in solution. (A) Amperograms of PAP. The potential was stepped from −0.1 V to 0.3 V at 10 s. (B) Current at 20 s vs. PAP concentration.

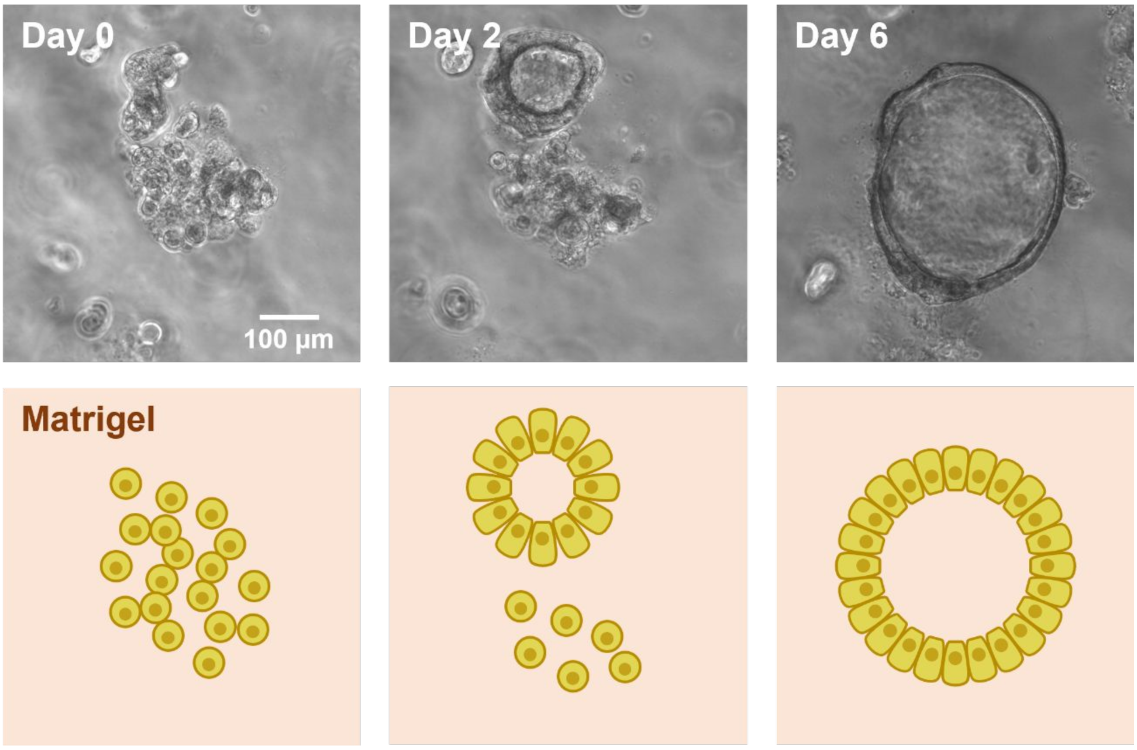

Fig. S3

Images and schematics of organoids cultured for 0, 2, and 6 days.

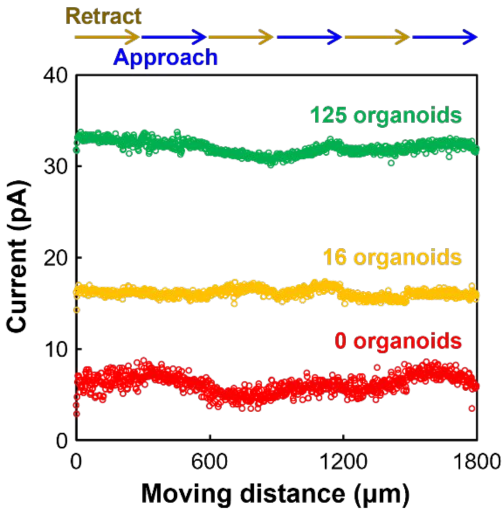

Fig. S4

SECM of ALP activity in 0, 16, and 125 organoids within hydrogel domes. Potential: 0.3 V. Culture period: 5 days. The graph shows the currents at moving distances.

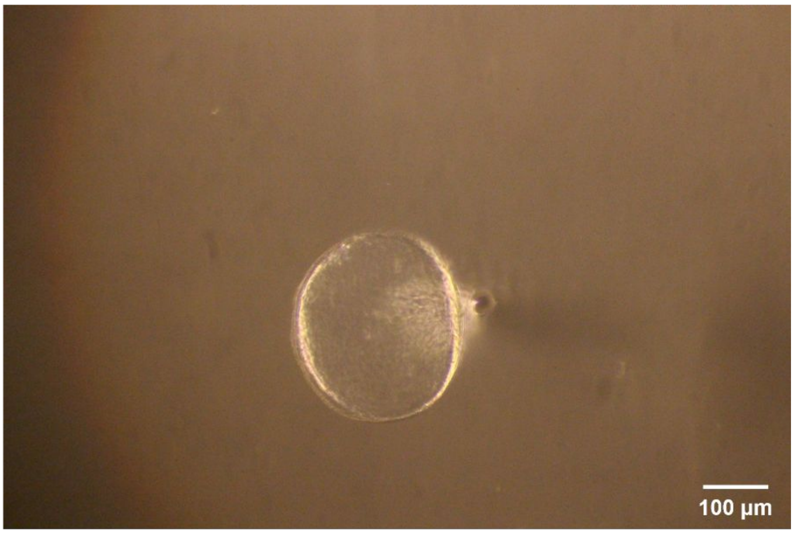

Fig. S5

Image of Fig. 4A before trimming.

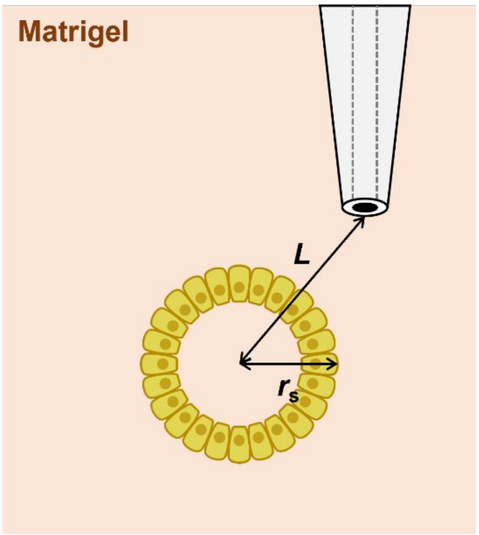

Fig. S6

Schematic for calculating PAP concentration. r_s: organoid radius. L: distance between the center of the organoid and the electrode.

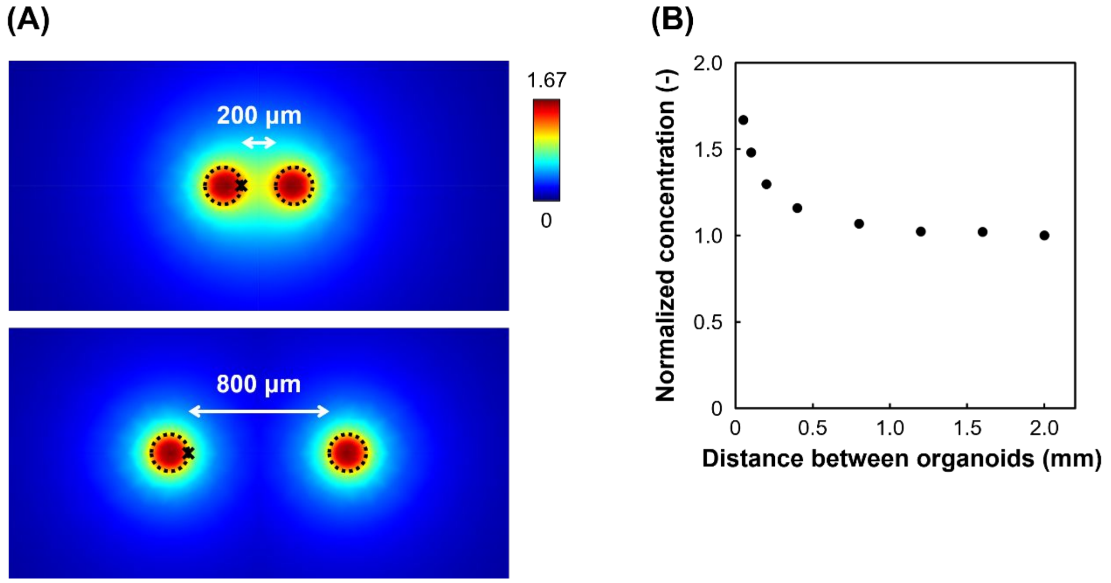

Fig. S7

Simulation of the effect of distance between organoids on enzymatic product concentrations. (A) Cross-section of normalized concentrations. The distances between organoids were 200 and 800 μm. (B) Effect of distance on the concentration at the position indicated by "X." Black dotted circles represent organoid positions. 3D simulation models were constructed using COMSOL Multiphysics software (ver. 5.4; COMSOL Inc., USA) to visualize diffusion. A time-dependent model was used. The product generation rate in the organoids was set to 1 mol/($m^3 \cdot s$). The initial concentration in the model was set to 0 mM (= 0 mol/m^3). The diffusion coefficient was set to 7.55×10^{-10} m^2/s. The concentration profile at 30 min was used for analysis. Two organoids with diameters of 200 μm were modeled, with distances between them of 50, 100, 200, 400, 800, 1600, and 2000 μm. Concentrations were normalized using the 2000-μm data.
